# Rhamnolipid-coated W/O/W double emulsion nanoparticles for efficient delivery of doxorubicin/erlotinib and combination chemotherapy

**DOI:** 10.1186/s12951-021-01160-4

**Published:** 2021-12-07

**Authors:** Yeeun Lee, Donghyun Lee, Eunyoung Park, Seok-young Jang, Seo Young Cheon, Seongryeong Han, Heebeom Koo

**Affiliations:** Department of Medical Life Sciences, Department of Biomedicine & Health Sciences, Catholic Photomedicine Research Institute, College of Medicine, The Catholic University of Korea, 222 Banpo-daero, Seocho-gu, 06591 Seoul, Republic of Korea

**Keywords:** Combination therapy, Double emulsion, Nanoparticle, Doxorubicin, Erlotinib, Rhamnolipid

## Abstract

**Background:**

Combination therapy using more than one drug can result in a synergetic effect in clinical treatment of cancer. For this, it is important to develop an efficient drug delivery system that can contain multiple drugs and provide high accumulation in tumor tissue. In particular, simultaneous and stable loading of drugs with different chemical properties into a single nanoparticle carrier is a difficult problem.

**Results:**

We developed rhamnolipid-coated double emulsion nanoparticles containing doxorubicin and erlotinib (RL-NP-DOX-ERL) for efficient drug delivery to tumor tissue and combination chemotherapy. The double emulsion method enabled simultaneous loading of hydrophilic DOX and hydrophobic ERL in the NPs, and biosurfactant RL provided stable surface coating. The resulting NPs showed fast cellular uptake and synergetic tumor cell killing in SCC7 cells. In real-time imaging, they showed high accumulation in SCC7 tumor tissue in mice after intravenous injection. Furthermore, enhanced tumor suppression was observed by RL-NP-DOX-ERL in the same mouse model compared to control groups using free drugs and NPs containing a single drug.

**Conclusions:**

The developed RL-NP-DOX-ERL provided efficient delivery of DOX and ERL to tumor tissue and successful tumor therapy with a synergetic effect. Importantly, this study demonstrated the promising potential of double-emulsion NPs and RL coating for combination therapy.

**Graphical Abstract:**

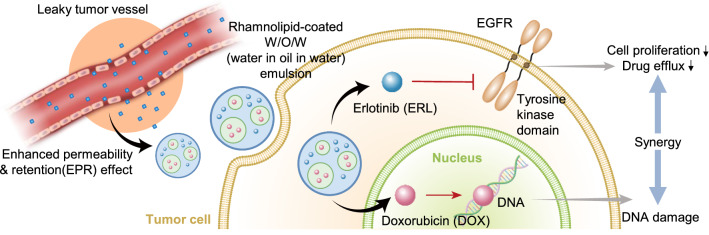

**Supplementary Information:**

The online version contains supplementary material available at 10.1186/s12951-021-01160-4.

## Introduction

Despite the extensive amount of time and effort devoted to the fight against cancer over many years, cancer remains a leading cause of death worldwide, with nearly 10 million deaths reported in 2020 [1]. In cancer therapy, chemotherapy has a long history as one of the primary treatments. In particular, beyond the traditional approach involving only a single drug, growing attention has been paid to various cocktail formulations consisting of two or more drugs [2]. Combination chemotherapy could modify multiple signaling pathways and mechanisms using more than one drug simultaneously. Various studies have been investigated combination therapy with numerous pairs of drugs including paclitaxel/cisplatin, paclitaxel/alendronate, gemcitabine/doxorubicin, doxorubicin/dexamethasone [3–6]. Some of them were synergetic and resulted in better clinical outcomes, but there were many cases that were less effective [7]. This highlights the importance of finding a synergetic combination of drugs [8]. Among them, doxorubicin (DOX) and erlotinib (ERL) have shown their pronounced synergy in many cases including glioblastoma, hepatocellular carcinoma, non-small cell lung cancer, and triple-negative breast cancer [9–12]. It has been reported that ERL inhibits epidermal growth factor receptor (EGFR) and makes tumor cells more sensitive to nucleotide damage induced by DOX [13]. In addition, ERL also inhibited P-glycoprotein pump-based drug efflux and increased the retention of other drugs inside tumor cells [14].

For successful therapy, efficient delivery of the drugs to the tumor tissue is critical. It can maximize the efficacy of chemotherapy and minimize the side effects associated with non-specific accumulation in normal organs [15]. Nanoparticles (NPs) have gathered attention from researchers as promising drug carriers [16]. They can solubilize hydrophobic drugs without aggregation in aqueous condition for injection into the human body by syringe [17]. They also prevent fast excretion of drug from the body and increase circulation time in the blood [18]. Moreover, in tumor therapy, intravenously injected NPs showed high accumulation in tumor tissue because of penetration through gaps in blood vessel walls from fast angiogenesis and delayed excretion due to ineffective lymphatic drainage [19]. This situation has been called the enhanced permeability and retention (EPR) effect, which has been a major advantage of NPs in tumor-targeted drug delivery [20].

When researchers incorporate more than one drug into NPs for combination therapy, the chemical properties of the drugs should be considered carefully [21]. In the case of DOX and ERL, DOX has a primary amine group and generally is used as a salt form with hydroxyl chloride, which makes it hydrophilic. However, the log P value of ERL was reported as 3.1, and it is poorly water-soluble [22]. This means that NPs need to have both hydrophobic and hydrophilic parts in their structures for successful physical loading of DOX and ERL. Oil in water (O/W) single emulsion and block-copolymer micelle are representative NP types for drug delivery, but they are not suitable for DOX and ERL because their cores are only hydrophobic. Therefore, we focused on water in oil in water (W/O/W) double emulsion NPs for loading and delivery of DOX and ERL simultaneously [23]. To the best of our knowledge, there has been no trial to apply double emulsion NPs for the combination of DOX and ERL, even though it is a traditional formulation. In addition, the surface of self-assembled NPs generally is coated with amphiphilic surfactant molecules, which play a pivotal role in size control and stability of NPs. Recently, we reported that rhamnolipid (RL), a biosurfactant originated from *Pseudomonas aeruginosa*, can be used in development of NPs for drug delivery [24]. That study is the first to show the favorable ability of RL as a coating material of NPs for intravenous injection. In addition, RL is environmentally friendly and suitable for mass production [25].

**Scheme 1:**
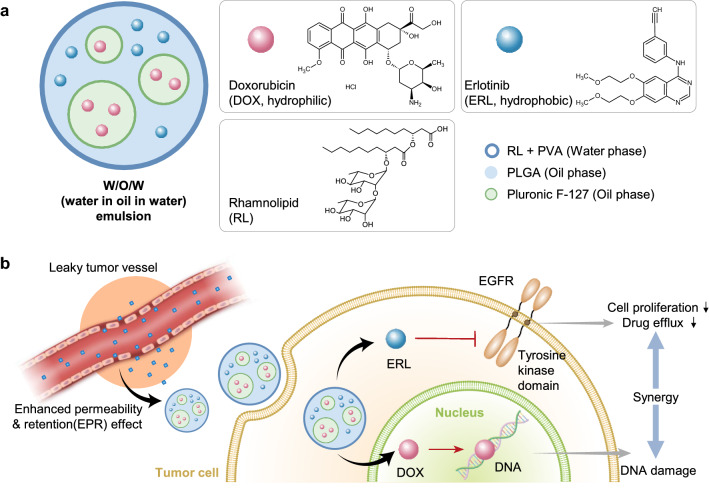
Schematic illustration of rhamnolipid-coated double-emulsion nanoparticles containing doxorubicin and erlotinib (RL-NP-DOX-ERL) for efficient drug delivery to tumor tissue and combination chemotherapy. **a** Structure of RL-NP-DOX-ERL formed by W/O/W double emulsion. **b** Drug delivery and combinational therapy using RL-NP-DOX-ERL

Based on this background, we developed RL-coated double emulsion NPs containing DOX and ERL (RL-NP-DOX-ERL) for efficient drug delivery to tumor tissue and combination chemotherapy (Scheme 1). The physicochemical properties of the developed NPs, encapsulation efficiency, and release pattern of the loaded DOX and ERL were analyzed in vitro. Their cellular uptake and tumor cell-killing effect were observed using SCC7, mouse squamous cell carcinoma cells. Then, SCC7-tumor-bearing mice were prepared, and the biodistribution of RL-NP-DOX-ERL and accumulation in the tumor site were monitored by real-time fluorescence imaging. In the same model, tumor suppression by RL-NP-DOX-ERL was analyzed after intravenous injection and compared with control groups.

## Methods

### Materials

Doxorubicin hydrochloride salt (DOX) and erlotinib free base (ERL) were obtained from LC Laboratories (Woburn, MA, USA). Rhamnolipid (RL) was purchased from AGAE Technologies (Corvallis, OR, USA). Poly(vinyl alcohol) (PVA), poly(d,l-lactide-*co*-glycolide) (PLGA), pluronic F-127, Tween 80, and sodium chloride (NaCl) were purchased from Sigma-Aldrich (Logan, UT, USA). Dimethyl sulfoxide (DMSO) was purchased form Samchun (Pyeongtaek, Gyeonggi-do, Korea). Dichloromethane (DCM) and Triton X-100 were obtained from Daejung Chemical Co. (Siheung, Gyeonggi-do, Korea). Glycerin was purchased from Junsei Chemical Co., Ltd (Tokyo, Japan). DiIC_18_(5) solid (1,1’-Dioctadecyl-3,3,3’,3’-Tetramethylindodicarbocyanine, 4-Chlorobenzenesulfonate Salt) (DiD) and Hoechst 33,342 were obtained from Invitrogen (Waltham, MA, USA). RPMI medium, Dulbecco’s phosphate-buffered saline (DPBS), and fetal bovine serum (FBS) were obtained from Biowest (Nuaille, France). Thiazolyl blue tetrazolium bromide (3-[4,5-dimethyl-thiazol-2-yl]2,5-diphenyltetrazolium bromide) (MTT), phosphate-buffered saline (PBS), 4% paraformaldehyde, and 10% formalin were obtained from Biosesang (Seongnam, Gyeonggi-do, Korea). Antibiotic-antimycotic solution and 0.05% Trypsin-EDTA were purchased from Gibco-BRL (Grand Island, NY, USA). Optimal cutting temperature (OCT) compound was purchased from Scigen Scientific, Inc. (Gardena, CA, USA).

### Preparation of nanoparticles

RL-NP-DOX-ER was prepared by the W/O/W solvent evaporation method with probe sonication. First, 2 mg DOX was dissolved in 250 μl distilled water (DW). This solution was added to 70 μl DMSO containing 2.5 mg ERL and 1.2 ml DCM containing 30 mg PLGA and 30 mg pluronic F-127. The mixture was emulsified by probe sonicator (VC505, Sonics Inc., Newtown, CT, USA) at 100 W for 1.5 min in an ice bath. Then, this W/O emulsion was added into 3.5 mL 0.25 wt% PVA aqueous solution containing 8.4 mg RL and 25 mg NaCl. The mixed solution was sonicated by probe sonicator at 200 W energy output for 1.5 min in an ice bath. DCM was evaporated by stirring at 80 °C for 40 min. The resulting NP solution was dialyzed (MWCO: 14 kda) in DW for 1 h to remove unloaded drugs. Control DOX-ERL-NPs were prepared using 0.5 wt% PVA aqueous solution without RL. We loaded 100 μg of DiD in oil phase for the particles used in animal imaging.

### Characterization of nanoparticles

The size and zeta-potential of the NPs were measured using Zetasizer Nano ZS90 (Malvern Instruments, UK). To measure the size and zeta potential of NPs, we diluted them with PBS ten-fold and analyzed them with Zetasizer. The morphology of NPs was observed by transmission electronic microscope (JEM1010, JEOL Ltd, Tokyo, Japan). Each NPs were diluted in DW with the concentration of 20 μg/ml of DOX and put on copper grid before imaging. The high-resolution image of RL-NP-DOX-ERL was obtained by cryo-TEM (Tecnai G2F20 Cryo, FEI, Netherlands). To observe size stability of RL-NP-DOX-ERL, we diluted the NPs 10-fold in PBS and PBS with 10% FBS for 1–3 week, and size of NPs was measured every day using Zetasizer. Encapsulation efficiency (EE) and release of DOX and ERL were measured in buffer solution (DMSO: PBS: DW = 5:4:1 including 1%(v/v) Triton X-100). The amounts of DOX and ERL were calculated by measuring fluorescence at Ex 490 nm/Em 570 nm and the absorbance at 342 nm using a microplate reader (Synergy H1 Hybrid Multi-Mode Reader, BioTek Inc., UT, USA). To evaluate the release of drugs, the NP solution was loaded into a dialysis bag (MWCO: 14,000 D) and dialyzed with 20 ml PBS with 2% (w/v) Tween 80 at room temperature. At each time point, external solution was collected and replaced with fresh PBS. Collected solution was measured in buffer solution (DMSO: PBS: DW = 5:4:1 including 1%(v/v) Triton X-100) to calculate the quantity of released DOX and ERL. The amounts of DOX and ERL were calculated by measuring fluorescence at Ex 490 nm/Em 570 nm and the absorbance at 342 nm using a microplate reader (Synergy H1 Hybrid Multi-Mode Reader, BioTek Inc., UT, USA).

### Cell viability assay

For the MTT assay, SCC7 tumor cells were seeded in a 96-well plate (5 × 10^3^cells/well) and incubated in RPMI for 24 h. Each drug and NP were added and incubated at 37 °C for 24 h. To evaluate combination effect, we tested free DOX and ERL based on the proportion of both drugs. To compare cytotoxic effect of NPs, we treated empty RL-NP, RL-NP-ERL, RL-NP-DOX, and RL-NP-DOX-ERL according to concentration. After 24 h, the solutions were replaced into fresh medium. Then, 20 μl MTT solution was added and incubated for 4 h. The absorbance of each well was measured at 450 nm by microplate reader. The combinational index (CI) value of DOX and ERL was calculated by Compusyn software.

### Cellular imaging

For imaging by fluorescence microscopy, SCC7 cells were seeded in a 24-well plate (5 × 10^3^ cells/well) and incubated in RPMI containing 10% FBS and 1% Antibiotic-Antimycotic (penicillin, streptomycin, and amphotericin B) at 37 °C for 2 days. After incubation with free DOX, RL-NP-DOX-ERL, and NP-DOX-ERL, the cells were treated with Hoechst 33342 (Ex/Em=352/451 nm) for cell nuclei staining for 20 min. After washing, the medium was replaced with phenol red-free RPMI. Fluorescent images were obtained using DAPI and rhodamine filter at the magnification of 400 times by fluorescence inverted microscope (IX71, Olympus, Tokyo, Japan). For imaging by confocal microscopy, SCC7 cells were seeded in a confocal dish (5 × 10^4^ cells/dish) and incubated in RPMI for 2 days. We incubated free DOX, RL-NP-DOX-ERL, and NP-DOX-ERL (4 μg/ml of DOX) for 2 h. Then, the cells were washed and incubated with Hoechst 33,342 for cell nuclei staining for 20 min. After fixation with 4% paraformaldehyde, the samples were washed and observed in fresh PBS by confocal laser scanning microscope (LSM800, Carl Zeiss, Germany).

For flow cytometry, SCC7 cells were seeded in a 24-well plate (5 × 10^3^cells/well) and incubated in RPMI containing 10% FBS and 1% antibiotics at 37 °C for 2 days. We incubated free DOX, RL-NP-DOX-ERL, and NP-DOX-ERL (4 μg/ml of DOX) for 2 h. After washing with PBS, cells were treated with trypsin for 3 min, and collected with FACS buffer (PBS with 5% (v/v) FBS) for centrifugation. Samples were centrifuged at 1500 rpm for 5 min, and the supernatant was removed. The cells were suspended with FACS buffer (PBS with 5% (v/v) FBS) and centrifuged again at 1500 rpm for 5 min. After removing supernatant, cells were suspended with 500 μl of FACS buffer (PBS with 5% (v/v) FBS) and transferred in FACS tube. To compare the amount of cellular uptake, cells were analyzed at PE-A fluorescence for the detection of DOX using flow cytometry (FACSCanto, BD Biosciences, Bedford, MA, USA).

### In vivo and ex vivo near-infrared fluorescence (NIRF) imaging

For the tumor allograft model, SCC7 cells (2 × 10^6^ cells) were inoculated subcutaneously into C3H/HeN mice. After the tumor grew to 150–00 mm^3^, free DiD or DiD-loaded NPs were injected by intravenous administration. Whole body distribution of each sample and NIRF intensity in the blood were observed at 1, 3, 6, 12, and 24 h after injection through IVIS Lumina XRMS (PerkinElmer Inc., Waltham, MA, USA) using DiD (Ex/Em = 660/710 nm) filter. At 24 h after injection, tumor and major organs including heart, lung, liver, spleen and kidney were excised and similarly observed using IVIS. Then, the excised tumors were embedded into OCT (optimal cutting temperature) compound and frozen at – 80 °C overnight. After cryo-section of the sliced tumor tissues and staining with Hoechst 33342, fluorescence images were obtained at the magnification of 100 times using an inverted fluorescence microscope.

### In vivo combination chemotherapy

To investigate the therapeutic effect of RL-NP-DOX-ERL, SCC7 cells (2 × 10^6^ cells) were inoculated subcutaneously into C3H/HeN mice. After the tumor size reached 50–100 mm^3^, each drug and NP (2.5 mg/kg of DOX) were injected intravenously four times, once every two days. Tumor size and body weight were measured every two days. After two weeks, the major organs and tumor were dissected and fixed in 10% formalin. The fixed tissues were sliced, stained by H&E (hematoxylin and eosin), and observed at the magnification of 100 times by bright field microscope (AxioImager A1, Zeiss, Germany).

### Statistical analysis

The statistical analysis was conducted with student t-test for two groups and one-way ANOVA for more than three groups. p values less than 0.05 were supposed to be statistically significant. The degree of significance was represented by asterisk (*p < 0.05, **p < 0.01 ***p < 0.001, ****p < 0.0001).

## Results

### Development and characterization of RL-NP-DOX-ERL

**Fig. 1:**
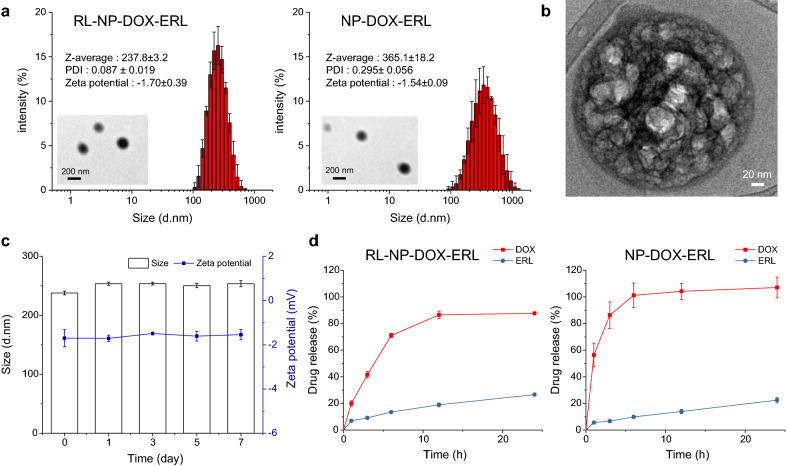
Characterization of RL-NP-DOX-ERL. **a** Size distribution and zeta potential of RL-NP-DOX-ERL (left) and NP-DOX-ERL (right) (n = 3). The inserted images were obtained by TEM. **b** Cryo-TEM image of RL-NP-DOX-ERL. **c** Size and zeta potential value of NP measured for 7 days (n = 3). **d** Time-dependent release of DOX and ERL from RL-NP-DOX-ERL (left) and NP-DOX-ERL (right) (n = 3)

For combination therapy, we developed RL-NP-DOX-ERL using the W/O/W solvent evaporation method to load hydrophilic DOX and hydrophobic ERL simultaneously. First, we prepared an O/W emulsion where DOX in the first water phase was loaded into an oil phase composed of ERL, PLGA (poly(d,l-lactide-*co*-glycolide)), and pluronic F-127. Then, this solution was re-emulsified by an outer water phase containing PVA (poly(vinyl alcohol)) and RL. To optimize the structure of RL-NP-DOX-ERL with small size, narrow size distribution, and high encapsulation efficiency (EE), we tested various compositions and conditions and found the optimal one, as shown in Additional file 1: Table S1. The optimized condition of RL-NP-DOX-ERL contained 2 mg of DOX and 3.5 mg of ERL as drugs, 30 mg of PLGA and pluronic F-127 as polymers used in oil phase, and 0.25 wt% PVA, 0.24 wt% RL, and 25 mg NaCl as stabilizer and surfactants in outer water phase. In this system, RL was used as a surfactant and shown to be suitable for stabilizing NPs and enabling prolonged blood circulation [24]. The size of RL-NP-DOX-ERL was 237.7 ± 0.9 nm with a narrow distribution (PDI = 0.087), which is smaller than that of NP-DOX-ERL (365.1 ± 19.2) without RL (p < 0.001). The smaller size of RL-NP-DOX-ERL is expected to be more suitable for the EPR effect and penetration in tumor tissue (Fig. 1a). Empty RL-NPs and RL-NP-DOX-ERL showed similar sizes, with no significant size changes during drug loading (p = 0.476) (Additional file 1: Fig. S1). Both NPs showed spherical morphology in TEM images. A high-resolution image of RL-NP-DOX-ERL obtained by Cryo-TEM showed a complex inner structure containing both drugs inside an oil droplet (Fig. 1b) [23]. In addition, the size and zeta potential of RL-NP-DOX-ERL were maintained for one week not only in PBS but also 10%(v/v) FBS solution, demonstrating the high stability of RL-coated NPs (Fig. 1c and Additional file 1: Fig. S2a). The size remained stable for 3 weeks (Additional file 1: Fig. S2b). In double-emulsion NPs, hydrophilic DOX and hydrophobic ERL should be encapsulated successfully for dual delivery. Importantly, in our RL-NP-DOX-ERL, the EEs of DOX and ERL are very high, about 92.7 ± 1.0 and 96.1 ± 5.5%, respectively. In drug release profiles, both DOX and ERL showed greater sustained release in RL-NP-DOX-ERL compared to NP-DOX-ERL without RL (Fig. 1d). We conclude that this system would be helpful for drug delivery by reducing drug loss during blood circulation of NPs.

To achieve simultaneous loading of hydrophilic and hydrophobic drugs, a self-assembled NP needs to have both hydrophilic and hydrophobic parts. Liposomes meet this criterion, with a hydrophilic aqueous core and hydrophobic membrane interface, and many studies have developed liposomes containing multiple drugs [26, 27]. Liposomes can be formed using various methods, with lipid film hydration being the most widely used. However, when a hydrophilic drug is dissolved in aqueous solution and loaded into the liposome from film, there is no driving force for drug molecules to move in. Therefore, a large amount of the drug remains in the solution outside of the liposome, which results in low EE [28]. To overcome this, the pH-gradient loading method was developed and provided high EE [29]. However, it has been applied to only a few drugs containing amine or carboxylic acid with suitable pKa value. On the other hand, in the first step of double emulsion method, the aqueous phase containing the hydrophilic drug was encapsulated entirely into the NP, so that high EE can be obtained for the hydrophilic drug as well as the hydrophobic one.

### Synergetic effect and the in vitro tumor cell-killing effect of RL-NP-DOX-ERL

**Fig. 2:**
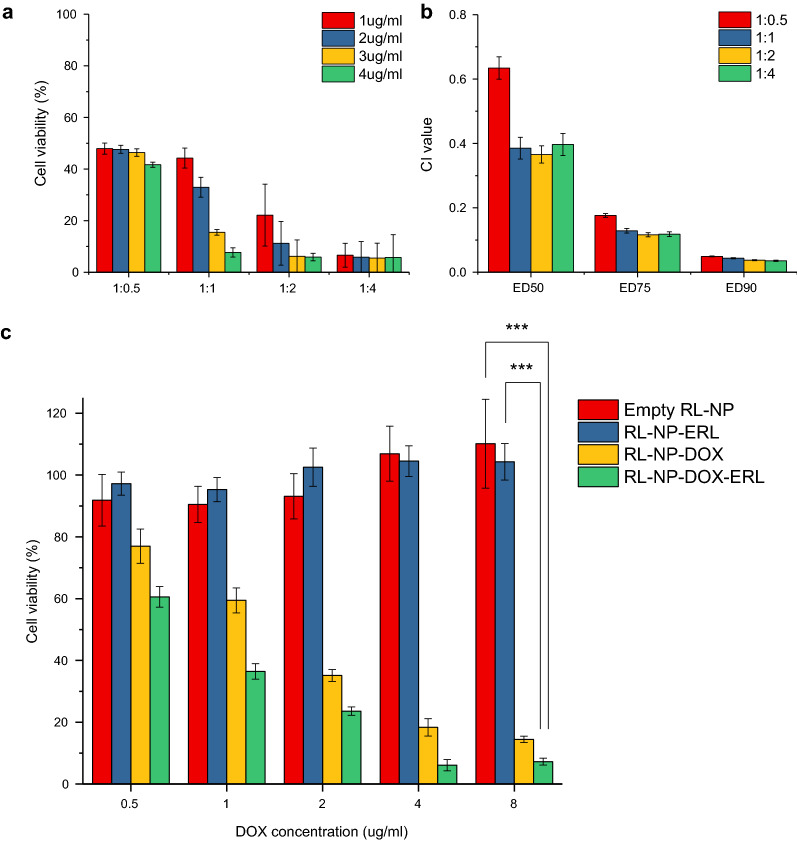
Synergetic effect between two drugs and the in vitro tumor cell-killing effect of RL-NP-DOX-ERL. **a** Cell viability after treatment with varying ratios of DOX and ERL (n = 6). **b** Combinational index (CI) values calculated based on data in **a**. **c** Cell viability after treatment with bare RL-NP, RL-NP-DOX, RL-NP-ERL, and RL-NP-DOX-ERL (n = 6)

For effective combinational therapy, we first evaluated the synergetic effect of free DOX and ERL according to composition ratio. They were administered to mouse squamous cell carcinoma (SCC7) cells at ratios ranging from 1:0.5 to 1:4 (DOX:ERL), and cell viability was evaluated by MTT assay (Fig. 2a). We observed dose-dependent death, indicating efficacy of both drugs. The synergy of combinational therapy can be evaluated using the combinational index (CI), where a value less than one indicates that the drugs are synergetic [8]. When we calculated CI values from the data, all ratios showed a CI value less than one, which indicates strong synergy of the drug combination (Fig. 2b) [30]. In particular, there was a relatively large difference between the drug ratio values of 1:0.5 and 1:1, so we used the 1:1 ratio as a target during formulation. Then, we administered RL-NP-DOX-ERL to SCC7 tumor cells and observed the cell viability (Fig. 2c). Empty RL-NP without drugs showed a viability greater than 90%, which indicated the biocompatibility of RL-NP itself. Most cells treated with RL-NP-ERL were alive, but RL-NP-DOX and RL-NP-DOX-ERL showed significant cytotoxicity (p < 0.001). Among them, RL-NP-DOX-ERL showed the strongest tumor cell-killing effect, demonstrating its potential for synergetic therapy through dual delivery of DOX and ERL.

### In vitro cellular uptake of RL-NP-DOX-ERL

**Fig. 3:**
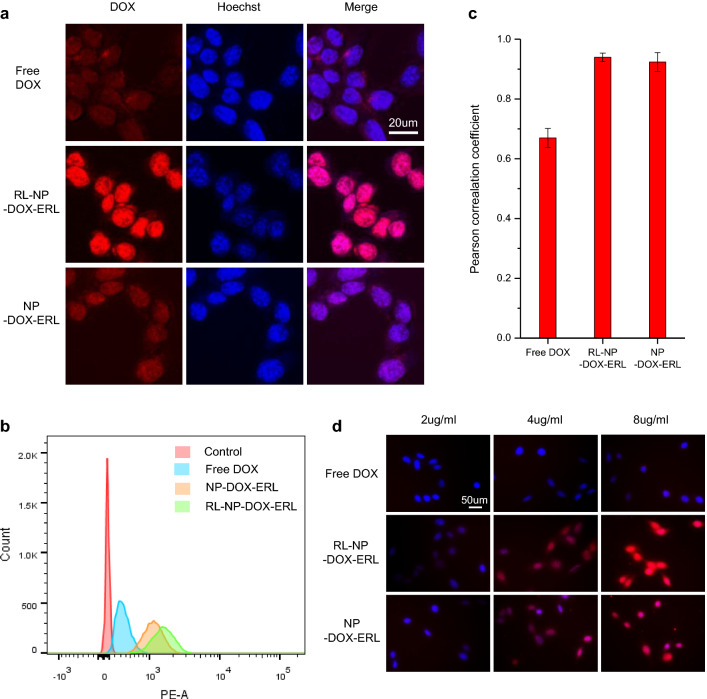
In vitro cellular uptake of RL-NP-DOX-ERL in SCC7 tumor cells. **a** Confocal microscopy images reveal cellular uptake of free DOX, NP-DOX-ERL, and RL-NP-DOX-ERL. Red and blue colors indicate DOX and Hoechst, respectively. **b** Flow cytometric analysis of SCC7 cells after treatment with free DOX, NP-DOX-ERL, and RL-NP-DOX-ERL. **c** Pearson correlation coefficients between the fluorescence signals from DOX and Hoechst in confocal images (***p < 0.001). **d** Fluorescent images of SCC7 cells after treatment with free DOX, NP-DOX-ERL, and RL-NP-DOX-ERL (different concentrations)

For accurate function of RL-NP-DOX-ERL in a tumor, RL-NP-DOX-ERL should be internalized in the tumor cell. Therefore, we observed cellular uptake of RL-NP-DOX-ERL in SCC7 cells by confocal microscopy and flow cytometry using the intrinsic fluorescence of DOX. In Fig. 3a, RL-NP-DOX-ERL showed a more intense red signal in SCC7 cells than did free DOX and NP-DOX-ERL without RL. A similar trend could be observed in flow cytometry data (Fig. 3b). These data showed fast cellular uptake of NPs after RL coating, and we think that inhibition of drug efflux by ERL might have contributed to the result. The antitumor effect of DOX is generated by intercalation into the nucleotide, so that DOX should move to the nucleus after cellular uptake. To analyze this in our experiment, we calculated Pearson correlation coefficients between the fluorescence signals from DOX and Hoechst in confocal images (Fig. 3c). All samples including free DOX, NP-DOX-ERL, and RL-NP-DOX-ERL showed high coefficients (over 0.6) with significant co-localization of DOX in the nucleus. This shows that DOX could move freely to the nucleus after cellular uptake, which is different from other cases where DOX was loaded into the hydrophobic part after desalting. All samples showed a dose-dependent increase in cellular uptake, and RL-NP-DOX-ERL showed a significantly higher intensity than did the other groups (p < 0.001 at 8 μ/ml) (Fig. 3d and Additional file 1: Fig. S3). The fast cellular uptake of RL-NP-DOX-ERL and retention of more DOX molecules inside tumor cells also were observed in time-dependent cell images, as in Additional file 1: Fig. S4. These results support the superior tumor cell-killing effect of RL-NP-DOX-ERL, demonstrated in Fig. 2.

### In vivo biodistribution of RL-NP-DOX-ERL

**Fig. 4:**
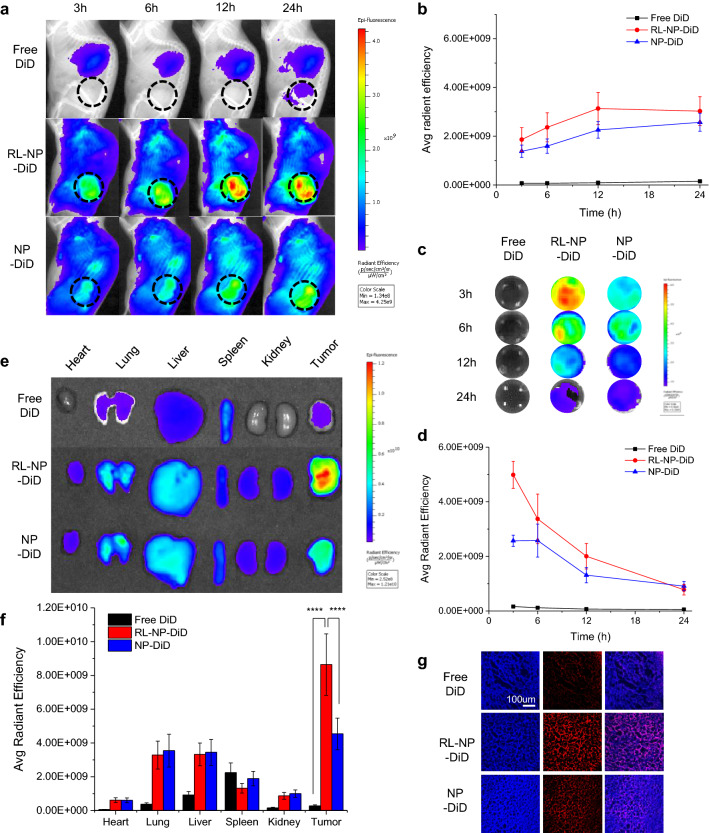
In vivo biodistribution of RL-NP loading DiD in the SCC7 allograft model (n = 3). **a** Whole body images of SCC7 tumor-bearing mice after intravenous injection of free DiD, RL-NP-DiD, and NP-DiD. Black dotted circles are tumor regions. **b** Near-infrared (NIR) fluorescence intensity in tumor area from **a**. **c** Fluorescence images of blood samples from mice. **d** NIR fluorescence intensity in blood from **c**. **e** Ex vivo NIR fluorescence images of tumors and major organs. **f** NIR fluorescence intensity in tumors and major organs in **e** (****p < 0.0001). **g** Fluorescence images of cryo-sectioned tumor tissues

Based on promising in vitro results, we observed the in vivo distribution of RL-NP-DOX-ERL in SCC7 tumor allograft mice. We used DiD (1,1′-Dioctadecyl-3,3,3′,3′-Tetramethylindodicarbocyanine, 4-Chlorobenzenesulfonate Salt), a near-infrared fluorescence dye after physical loading into RL-NPs for in vivo imaging. Its biodistribution was observed by real-time imaging using IVIS imaging system (Fig. 4a). The absorption spectra of DiD show maximum value around 650 nm, and the Ex/Em = 660/710 nm filter set was selected and used as an optimal one based on mouse images (Additional file 1: Fig. S5). As we expected, the tumor site grew brighter after intravenous injection of RL-NP-DiD, showing high accumulation in the tumor tissue. On the other hand, free DiD showed negligible fluorescence, which might be due to aggregation and excretion from the body. The NIR fluorescence signal in the tumor tissue of RL-NP-DiD was slightly higher than that of NP-DiD without RL (Fig. 4b). RL-NP-DiD showed relatively stable blood circulation, which is suitable for the EPR effect (Fig. 4c and d). At 24 h following injection, the mice were sacrificed, and tumors and major organs containing heart, lung, liver, spleen and kidney were analyzed. The higher accumulation of RL-NP-DiD than other groups was clear in extracted tumors, showing that RL on the surface of NP is helpful for drug delivery to tumor tissue (p < 0.0001) (Fig. 4e, f). The tumor tissues of RL-NP-DiD showed 31.3- and 1.9-fold higher NIR fluorescence intensity than those of free DiD and NP-DiD, respectively. Sliced tumor tissue also showed a similar trend and the strongest signal in the case of RL-NP-DiD (Fig. 4g). Based on these results, we conclude that RL-NP could provide long blood circulation and high accumulation in tumor tissue by the EPR effect after intravenous injection, likely because RL stably maintains the size of NPs in the blood and fast cellular uptake after arrival in tumor tissue. In addition, real-time in vivo imaging data and high accumulation of RL-NP in tumor tissue showed its potential in image-guided drug delivery which enables simultaneous monitoring of the delivered drug or diagnosis of disease [31–33].

The surface property of the NP is important for its stability, blood circulation, and cellular uptake. For surface coating, we used RL, an amphiphilic biosurfactant with one carboxyl and five hydroxyl groups in its chemical structure, which has been used widely in agriculture, cosmetics, and pharmaceutics [25]. Along with its advantages of eco-friendly synthesis and facile mass production, we expected that it could provide favorable stability in blood circulation while not inhibiting cellular uptake; this was partially supported in the cell and animal data in this study. We tried not to used polyethylene glycol for surface coating because it can reduce cellular uptake due to its strong anti-fouling effect [34]. As we expected, RL-NP-DOX-ERL showed faster uptake and more effective retention inside the cell than did free DOX, while enabling long circulation after intravenous injection.

### In vivo anti-tumor effects of RL-NP-DOX-ERL

**Fig. 5:**
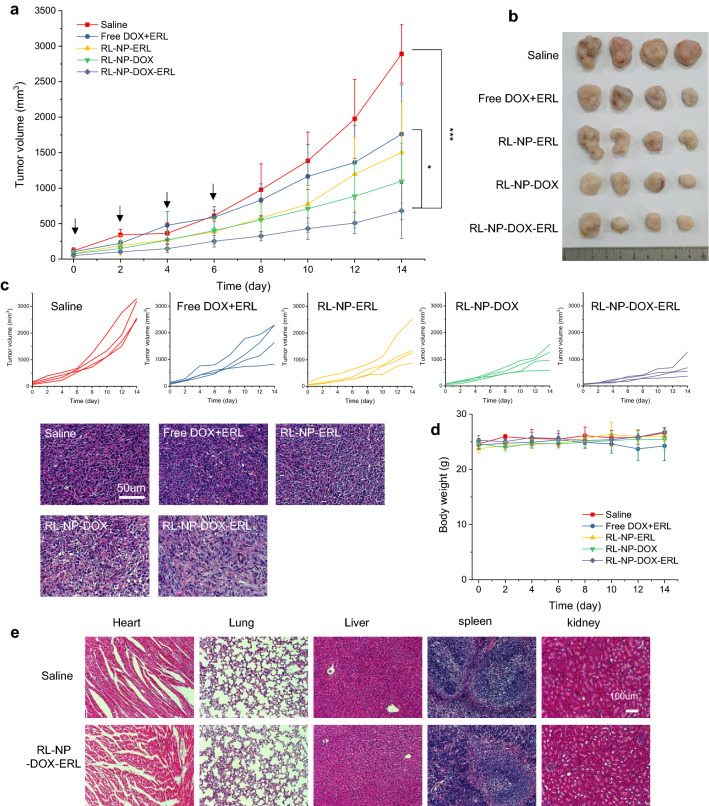
Therapeutic result of RL-NP-DOX-ERL in the SCC7 allograft model (n = 4). **a** Tumor growth graph of the SCC7 allograft model injected with saline, free DOX and ERL, RL-NP-ERL, RL-NP-DOX, and RL-NP-DOX-ERL (*p < 0.05, ***p < 0.001). Black arrows mean the intravenous injection of samples. **b** Tumor images from the mice sacrificed after 2 weeks. **c** H&E-stained images of the sliced tumor tissues. **d** Body weights of the mice measured for 2 weeks. **e** H&E-stained images of the sliced major organs

Finally, we evaluated the effect of RL-NP-DOX-ERL in combinational chemotherapy in SCC7 allograft mice. Each sample was injected intravenously with the 2.5 mg/kg DOX concentration every 2 days (a total of four times). The groups injected with free drugs showed less tumor suppression compared to the NP-injected groups (Fig. 5a). Among NPs, RL-NP-DOX-ERL suppressed the tumor successfully, which was 2.2- and 1.6-fold more effective compared to RL-NP-ERL and RL-NP-DOX, respectively. This result suggested that RL-NP-DOX-ERL showed a synergetic effect between DOX and ERL for chemotherapy under in vivo conditions compared to single drug-loaded NPs. In images of excised tumors, tumors treated with RL-NP-DOX-ERL showed the smallest size among all groups (Fig. 5b). An H&E image of the sliced tumor tissue revealed that the RL-NP-DOX-ERL-treated tumor was destroyed more than the others, which represented the superior therapeutic effect of the treatment (Fig. 5c). In all groups, significant alteration of body weight was not observed during therapy, which means there was no severe systemic toxicity (Fig. 5d). H&E (hematoxylin and eosin) images of the major organs of RL-NP-DOX-ERL-treated mice did not show a significant difference compared to those of the saline-treated control group, showing its biocompatibility (Fig. 5e). These data demonstrated that RL-NP-DOX-ERL enabled successful combination therapy in mice by efficient delivery of DOX and ERL and their synergetic effect.

Recent papers reported that the sequence of drug treatment is important and faster release of ERL is helpful in combination of DOX and ERL [13, 35, 36]. The cell lines used in the studies include triple negative breast cancer cell BT-20 cells, HER-2 overexpressing MDA-MB-453 cells, luminal MCF7 cells, and Hs578Bst which have severe resistance against DOX. ERL can increase sensitivity to DNA-damaging agents like DOX by reprogramming signaling pathway involved in tumor growth, so that pre-treatment of ERL can be highly effective in such cases. However, SCC7 cells used in our experiments was relatively more sensitive to DOX than those cell lines. During tests of the combination of DOX and ERL in SCC7 cells, lower cell viability was observed when we treated DOX first and ERL later compared to the opposite case, which is different with the results in other papers (Additional file 1: Fig. S6). Moreover, the group treated with DOX and ERL simultaneously also showed lower viability at similar level with that treated with DOX first. In addition, we injected our NPs four times in mice experiment, so that we expect that the ERL injected at initial stage could increase the drug sensitivity to DOX injected later. Therefore, sequential drug delivery would be particularly important in the cases with resistant cell lines or single injection to body.

## Conclusions

In summary, we developed rhamnolipid-coated double emulsion nanoparticles containing doxorubicin and erlotinib (RL-NP-DOX-ERL) and applied them to combinational tumor therapy. Double emulsion-type NPs enabled simultaneous loading of hydrophilic DOX and hydrophobic ERL in a single NP. RL, an amphiphilic biosurfactant, provided a relatively more stable surface coating than control NP with PVA only. The ratio between DOX and ERL was optimized based on CI values during cell viability tests. The size of the resulting RL-NP-DOX-ERL was about 237.7 ± 0.9 nm, and both DOX and ERL were loaded with high EE values of 92.7 and 96.1%, respectively. The system showed fast cellular uptake and a synergetic effect of the drug combination in killing SCC7 tumor cells. In SCC7 tumor-bearing mice, RL-NP-DOX-ERL showed 31.3- and 1.9-fold higher fluorescence intensity in tumor tissue compared to cases of free DiD dye and control NPs without RL, respectively, after intravenous injection. Successful tumor suppression in the same model demonstrated the promising potential of RL-NP-DOX-ERL for efficient drug delivery and combination therapy with a synergetic effect between multiple drugs.

## Supplementary Information

**Additional file 1.** Supplementary table and figures.

## Data Availability

All data generated or analyzed during this study are included in this published article.

## Abbreviations

PDT: Photodynamic therapy
NP: Nanoparticle
RL: Rhamnolipid
DOX: Doxorubicin
ERL: Erlotinib
PLGA: Poly(d,l-lactide-*co*-glycolide)
PVA: Poly(vinyl alcohol)
EPR: Enhanced permeability and retention
MTT: 3-[4,5-dimethyl-thiazol-2-yl]-2,5-diphenyltetrazolium bromide
H&E: Hematoxylin and eosin
