# Supplementary material for: Rhamnolipid-coated W/O/W double emulsion nanoparticles for efficient delivery of doxorubicin/erlotinib and combination chemotherapy
Source: J Nanobiotechnology. 2021 Dec 7;19:411. doi: 10.1186/s12951-021-01160-4 (PMC8650405; doi:10.1186/s12951-021-01160-4)
Supplement: Supplementary file 1 — Additional file 1. Supplementary table and figures. [file 12951_2021_1160_MOESM1_ESM.docx]

**Supplementary information**

**Rhamnolipid-coated W/O/W double emulsion nanoparticles for efficient delivery of doxorubicin/erlotinib and combination chemotherapy**

Yeeun Lee, Donghyun Lee, Eunyoung Park, Seok-young Jang, Seo Young Cheon, Seongryeong Han, Heebeom Koo^*^

Department of Medical Life Sciences, **Department of Biomedicine & Health Sciences, and** Catholic Photomedicine Research Institute, College of Medicine, The Catholic University of Korea, 222 Banpo-daero, Seocho-gu, Seoul, 06591, Republic of Korea

* Corresponding author. E-mail: [hbkoo@catholic.ac.kr](mailto:hbkoo@catholic.ac.kr)

| W1 | O | | | W2 | | | Mean particle size  (nm) | Polydispersity  index | Encapsulation  Efficiency  (%) | |
| --- | --- | --- | --- | --- | --- | --- | --- | --- | --- | --- |
| DOX  (mg) | ERL  (mg) | PLGA  (mg) | Pluronic  F-127  (mg) | PVA  (w/v %) | Rhamnolipid  (w/v %) | NaCl  (mg) |  |  | DOX | ERL |
| 2 | 3.5 | 30 | 30 | 2.5 | 0.24 | 25 | 224 | 0.383 | 75.8 | 100.5 |
| 2 | 3.5 | 30 | 30 | 1 | 0.24 | 25 | 225.3 | 0.266 | 71.8 | 71.9 |
| 2 | 3.5 | 30 | 20 | 1 | 0.24 | 25 | 209.6 | 0.241 | 91.0 | 92.9 |
| 2 | 3.5 | 20 | 30 | 1 | 0.24 | 25 | 223.3 | 0.332 | 96.1 | 79.4 |
| 2 | 3.5 | 30 | 30 | 1 | 0.5 | 25 | 197.7 | 0.261 | 81.1 | 76.3 |
| 2 | 3.5 | 30 | 30 | 0.25 | 0.24 | 25 | 242.6 | 0.177 | 78.4 | 76.9 |
| 2 | 3.5 | 30 | 30 | 0.25 | 0.5 | 25 | 221.1 | 0.241 | 84.2 | 84.4 |
| 2 | 2.5 | 30 | 30 | 0.25 | 0.24 | 25 | 237.8 | 0.087 | 92.7 | 96.1 |
| 2 | 2.5 | 30 | 30 | 0.5 | - | 25 | 365.1 | 0.295 | 38.9 | 91.3 |

**Table S1.** Conditions and characterization of RL-NP-DOX-ERL with varied composition. Selected one is underlined.

**Fig S1.** Size distributions of RL-NP-DOX, RL-NP ERL, and bare RL-NP (n=3).

**Fig S2.** Stability of RL-NP-DOX-ERL. (a) Size of NP measured in PBS containing 10%(v/v) FBS (n=3). (b) The size of NP measured in PBS for 3 weeks (n=3).


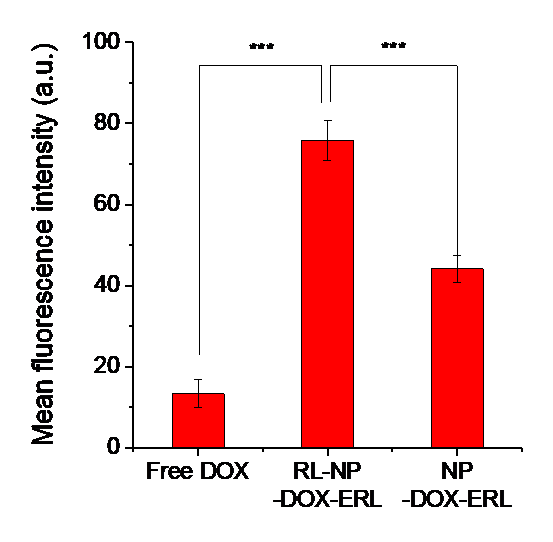


**Fig S3.** Mean fluorescence intensity of the samples in Fig. 3d at 8ug/ml (***p<0.001).

**Fig S4.** Fluorescent images of SCC7 cells after treatment with free DOX, NP-DOX-ERL, and RL-NP-DOX-ERL (different incubation times).

**Fig S5.** Optimization of filter set for DiD dye and *in vivo* imaging. (a) Absorption spectra of DiD dye. (b) Whole body images of mice after injection of RL-NP-DiD. Images were obtained using different filter sets.

**Fig S6.** Cell viability test using sequential treatment in SCC7 tumor cells. The interval of treating time is 24 h. (a) Cell viability after treatment of free drugs with different sequence (n=6). (b) Cellular viability after treatment of NPs with different sequence (n=6).
